# Efficient high-harmonic generation from a stable and compact ultrafast Yb-fiber laser producing 100 μJ, 350 fs pulses based on bendable photonic crystal fiber

**DOI:** 10.1007/s00340-016-6620-8

**Published:** 2017-01-11

**Authors:** James S. Feehan, Jonathan H. V. Price, Thomas J. Butcher, William S. Brocklesby, Jeremy G. Frey, David J. Richardson

**Affiliations:** 1 Optoelectronics Research Centre, University of Southampton, Hampshire, SO17 1BJ UK; 2 Department of Chemistry, University of Southampton, Hampshire, SO17 1BJ UK; 3 Present Address: Central Laser Facility, Rutherford Appleton Laboratory, Didcot, Oxfordshire, OX11 0QX UK

**Keywords:** Pulse Energy, Photonic Crystal Fiber, High Pulse Energy, Output Pulse Energy, Coherent Diffractive Imaging

## Abstract

The development of an Yb^3+^-fiber-based chirped-pulse amplification system and the performance in the generation of extreme ultraviolet (EUV) radiation by high-harmonic generation is reported. The fiber laser produced 100 μJ, 350 fs output pulses with diffraction-limited beam quality at a repetition rate of 16.7 kHz. The system used commercial single-mode, polarization maintaining fiber technology. This included a 40 μm core, easily packaged, bendable final amplifier fiber in order to enable a compact system, to reduce cost, and provide reliable and environmentally stable long-term performance. The system enabled the generation of 0.4 μW of EUV at wavelengths between 27 and 80 nm with a peak at ~45 nm using xenon gas. The EUV flux of ~10^11^ photons per second for a driving field power of 1.67 W represents state-of-the-art generation efficiency for single-fiber amplifier CPA systems, corresponding to a maximum calculated energy conversion efficiency of 2.4 × 10^−7^ from the infrared to the EUV. The potential for high average power operation at increased repetition rates and further suggested technical improvements are discussed. Future applications could include coherent diffractive imaging in the EUV, and high-harmonic spectroscopy.

## Introduction

The development of cladding-pumped fiber technology over the past two decades [1] has allowed for continuous wave (CW) powers above a kilowatt in single-mode fiber [2] and pulsed systems with gigawatt peak powers [3]. Nonlinearity is the biggest challenge for pulsed fiber lasers, and this is particularly true for femtosecond systems because the pulse quickly degrades due to self- and cross-phase modulation as the pulse energy is increased. However, there are a wide range of applications for femtosecond pulses that require modest energies and where users would benefit significantly from the availability of compact and practical sources with the ability to readily provide for repetition rate and average power scaling. Thermal management problems in traditional crystal-based femtosecond laser sources have until now hindered significant average power scaling, but fiber amplifiers have both high efficiency and an almost ideal geometry for thermal management. Furthermore, the cladding-pumping geometry that has enabled multi-kW class continuous wave fiber lasers should also enable scaling of the repetition rate and average power at reasonable cost based on well-established techniques, making femtosecond fiber-based chirped-pulse amplification (CPA) research important for future higher repetition rate sources [1, 3]. Examples of applications that would significantly benefit from such developments include femto-chemistry, materials processing, and even fundamental strong-field physics envisioned in future large scientific facilities [4].

The highest pulse energy from femtosecond fiber amplifiers has been achieved using large diameter and rigid rod-type fibers as these enable the largest guided-mode areas and hence minimize nonlinearities. Although the mode area is necessarily reduced when bending a fiber (due to the stress-induced increase in refractive index), using more conventional bendable fibers for the final amplifier enables a smaller package size and can provide technical advantages such as truly single-mode, single polarization operation. Conventional bendable fibers are also lower cost than rod-type fibers, which could be important both for industrial marking and cutting systems, and for lasers used for advanced imaging techniques [5, 6]. Furthermore, the launch of a recently commercialized pump combiner for a bendable large-mode area photonic crystal fiber indicates that systems based on this technology can be fully fiberized to improve stability compared with systems using free-space signal and pump coupling optics. Advancing the performance of bendable large-mode area fibers has also led to an active research community working in parallel with those developing rod-type fibers [7].

While there have been a number of fiber CPA systems based on rod-type fiber technology [8, 9] which are capable of high-harmonic generation (HHG) and coherent diffractive imaging [10] there have been far fewer reports of bendable fiber systems for these applications. (The main exception involved the use of an enhancement cavity to enable the EUV generation [11]). Motivated by the inherent benefits of maintaining a bendable and fully polarization maintaining (PM) fiber-based architecture, an investigation into whether a femtosecond CPA system based on this technology would provide the performance required for coherent extreme ultraviolet (EUV) radiation via HHG is presented here. As reported below, the system was highly stable and showed amplitude fluctuations of just 1% over long time periods for 100 μJ, 350 fs pulses at a repetition rate of 16.7 kHz. Nonlinear pulse distortion limited further pulse energy scaling. The measured *M* ^2^ of 1.07 also compares favorably with the *M* ^2^ = 1.1–1.2 typically achieved from rod-type fiber systems. As the compressor grating used a dielectric instead of a metallic coating the repetition rate is scalable and average power levels could readily be increased to at least 100 W if desired [12].

The main benefit of fiber systems is typically viewed as the ability to power scale using MHz repetition rates as we have reported earlier in a 150 W prototype version of our system [12] and >800 W is currently the state of the art from a single-branch fiber laser in the fs regime [13], the novelty in the current manuscript is how we have improved the architecture and performance in terms of both pulse energy, beam quality, and the fraction of the energy in the peak of the pulse (compared to the pre- and post-pulse pedestal). Ti:Sapphire lasers produce higher pulse energies than fiber systems, but the pulse quality in relation to spatial chirp and pulse front tilt, which are common and unwanted characteristics in bulk laser CPA systems and which are hard to measure, are absent in our system. The physical process of HHG is a stringent test of pulse temporal quality and spatial quality because the efficiency rapidly reduces for when the pulses display imperfections. By comparing this result with a wide range of state-of-the-art HHG sources, we show that this conversion efficiency is approximately 1.17 times greater than comparable systems using a single rod-type fiber [14, 15]. However, it is important to note our ±50% uncertainty window on XUV flux values so the ratio could be in the range from 2/3 to 3/2 times this value. Hence, while we fairly report that the central estimate is that our generation is at least as good as any reported to date, this central estimate may not contradict an expectation that high pulse energy systems would have higher efficiency if the lower estimate is taken. This improvement in XUV flux is attributed to the high spatial and temporal beam quality of the FCPA system. Other novel features reported are the use of a gas cell for the XUV generation as opposed to a gas jet, and how the excellent long-term laser stability permitted careful experimental optimization of the beam diameter/focusing-geometry, gas pressure, and position of the focal volume within the gas cell using the real-time feedback available from the in-house XUV diagnostics. In addition, the comparison of our results with other systems constitutes the first comprehensive review of generation efficiency in FCPA-driven HHG to span a broad range of fiber technologies and sources.

This paper is structured as follows. Section 2 describes the experimental setup of the fiber CPA system and then the performance of the system is reported in Sect. 3. The EUV generation is described in Sect. 4. A discussion of the EUV generation efficiency in comparison with rod-type systems is provided in Sects. 5, and 6 that contains a brief summary and conclusion.

## Fiber CPA system

**Fig. 1:**
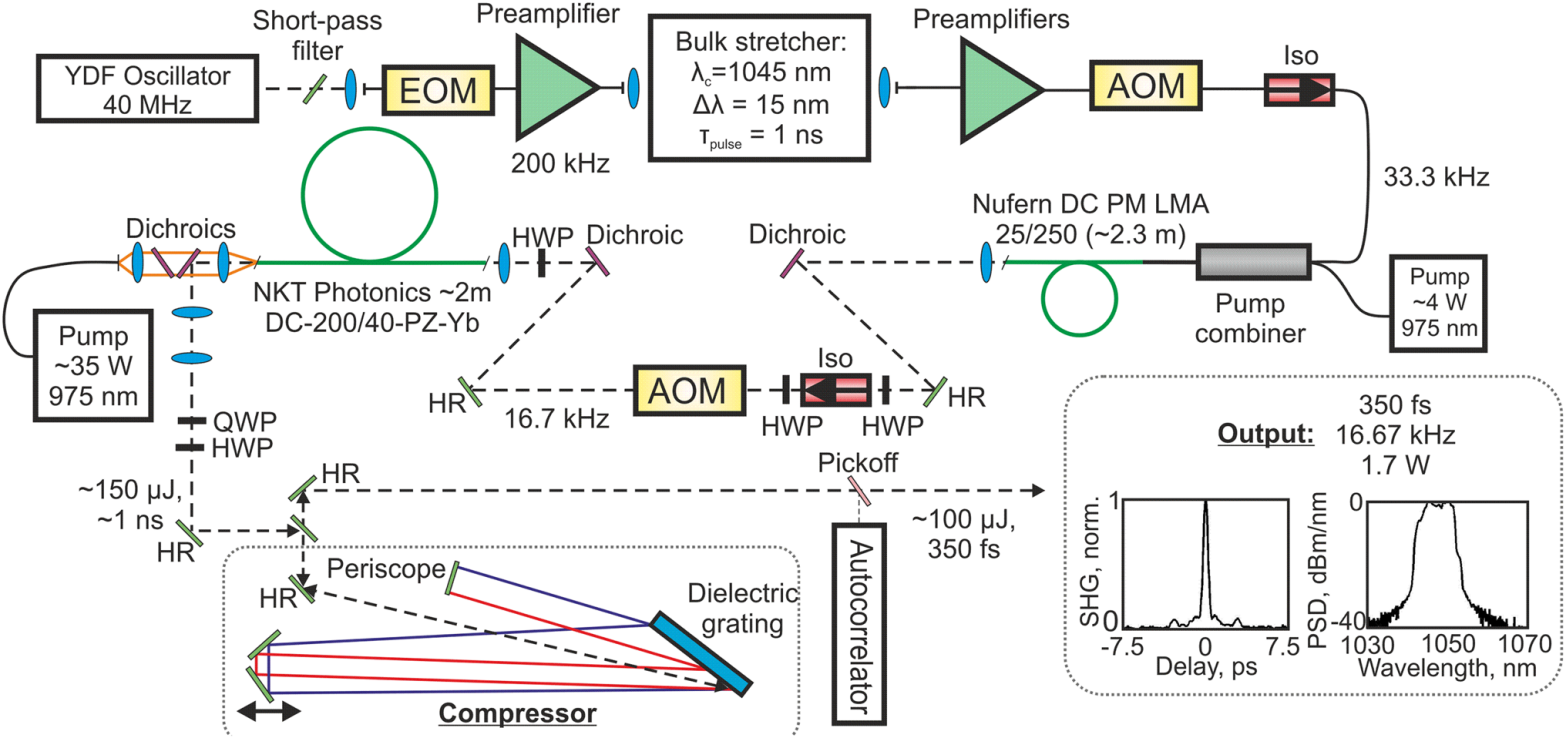
Schematic of the fiber CPA system. The system produced 350 fs, 100 μJ pulses with a peak power of 240 MW and an optical signal-to-noise ratio of 14 dB (measured in the spectral domain). *Inset* Autocorrelation and spectral data

Figure 1 shows a schematic of the fiber CPA system, which was fully fiberized from the stretcher output through to the final amplifier.

The seed was a mode-locked Yb-doped fiber-stretched pulse ring cavity oscillator similar to those reported in Refs. [16, 17], producing pulses with a central wavelength of 1064 nm and a bandwidth of 72 nm at the −10 dB level at 40 MHz repetition rate. The 0.75 nJ pulses were positively chirped (3 ps, compressible to 50 fs with second-order dispersion compensation). A 1053-nm hard-edged short-pass filter was used to cut down the bandwidth in order to concentrate energy extraction from the amplifiers in the wavelength span transmitted through the stretcher and compressor.

A fiberized EOM reduced the repetition rate to 200 kHz as this was found to give a good balance between gain and optical signal-to-noise ratio (OSNR) in the preamplifiers. The DC bias voltage applied to the EOM was controlled by a feedback loop (not shown in Fig. 1) which continuously maximized the extinction ratio to prevent thermally related drifts, which was critical for obtaining sufficient pulse energy from the preamplifiers to saturate the power amplifiers. Feedback was provided by a 20 dB tap in the preamplifier chain connected to a photodiode.

Following the EOM, a 975-nm core-pumped booster amplifier was used to maintain good OSNR after the 200-fold repetition rate reduction. A grating-based stretcher followed, and this had a standard design described in detail in Ref. [18] and was used previously in a similar fiber CPA system producing 100 µJ pulses with a comparable compressed duration [19]. The stretched pulse duration was 1 ns at the intensity FWHM, and the stretcher transmission window was 14 nm (edge-to-edge) with a central wavelength of 1045 nm, chosen for high gain per unit length in the final amplifier with minimal gain narrowing. The preamplifiers were 975 nm forward pumped using telecommunications-grade diodes and wavelength division multiplexed (WDM) pump couplers. Each preamplifier had an optical isolator at the input to prevent counter-propagating ASE from either depleting the gain or from damaging the preceding preamplifiers. Typically, the preamplifiers used a 2 m length of Yb-doped fiber, which was found to be optimal for achieving low noise and low nonlinearity while matching the gain bandwidth to the signal.

The first power amplifier was constructed using 2.3 m of Nufern DC 25/250 Yb-fiber (NA = 0.06 and 0.46 for the core and pump cladding, respectively) and was forward pumped with a fiberized 4 W, 975 nm diode using a fiberized multimode pump combiner with single-mode signal throughput. The fiber was coiled to a bend radius of 4 cm to maintain both fundamental mode operation and a small footprint for the amplifier (approximately 20 × 20 cm including the pump diode housing). The output end was polished to an angle of 5° to avoid optical feedback then a short-pass dichroic filter was used to remove residual pump light. A free-space AOM and isolator removed unwanted ASE between pulses and in the reverse direction, and also cut the repetition rate to 16.7 kHz. A half waveplate was used to maximize the AOM diffraction and a short-pass dichroic mirror removed short-wavelength ASE from the signal and residual backward propagation pump. Launch optics were water-cooled for long-term stability.

The final amplifier used a 2 m length of commercially available, single-mode, single polarization Yb-doped photonic crystal fiber (PCF) (NKT Photonics DC-200/40-PZ-Yb) with a core diameter of 40 μm and an effective mode field area of 760 μm^2^. The amplifier had angle-polished input and output facets to prevent feedback. The PCF was bent to a radius of 20 cm to provide polarizing operation and was backward pumped using a 35 W, 975 nm diode. This amplifier had a footprint of approximately 40 × 40 cm. Due to an initial period of use at high power in a previous experiment this particular fiber had suffered some photodarkening [20] and the associated excess losses resulted in the low overall conversion efficiency of approximately 11% (compared to launched pump power) observed here. Despite this pre-existing photodarkening-related loss, the fiber operation is now highly stable, as illustrated by the long-term power data shown in Fig. 4. Photodarkening is not an inherent limitation associated with the PCF structure, and it has been reduced in the current version of this fiber, which provides for pump to signal conversion efficiencies of 50–70%.

The amplifier output was collimated to 3.3 mm (1/e^2^ intensity radius) for transmission through the dielectric grating compressor [12]. The grating was 35 cm wide with a groove density of 1740 lines/mm and the four pass system had an edge-to-edge spectral window of 12 nm, transmission efficiency of 65%, and polarization extinction ratio of 27 dB.

## Fiber CPA performance

Key challenges when designing the preamplifiers were preserving the broad pulse bandwidth and maintaining a high OSNR because cascaded amplifiers can easily lead to both gain narrowing and significant ASE build up. The preamplifier chain had an output OSNR of 10 dB as measured from the spectrum, i.e., including ASE arriving between pulses in the time domain. A fiberized AOM and isolator then cut the repetition rate to 33.3 kHz and increased the OSNR to approximately 40 dB (by removing ASE between pulses) before transmission into the first power amplifier. The preamplifier output pulse energy was 90 nJ, which was reduced to 30 nJ at the input to the first power amplifier due to the AOM, isolator and pump combiner insertion losses. The first power amplifier used a fiber bend radius of 5 cm to achieve a single-mode output beam with *M* ^2^ = 1.1. It produced ~3 μJ output pulses with 23 dB OSNR before the following AOM diffraction reduced the pulse energy to 1.5 μJ and increased the OSNR to 30 dB. The final amplifier output pulse energy was 155 μJ (21.8 dB gain after the input coupling losses) with OSNR of 14 dB. The OSNR at the output of the compressor increased to 30 dB because only the 12 nm window of the signal spectrum is transmitted and the diffraction efficiency reduced the pulse energy to 100 μJ.

As is commonly the case with fiber systems, nonlinearity, not energy storage, limited the maximum pulse energy from the power amplifier. We calculated the saturation energy at a wavelength of 1045 nm using typical parameters for alumino-silicate fiber [21] to be 265 μJ for this amplifier, giving E/E_sat_ = 0.56. Saturation-related gain shaping was therefore not expected to be significant.

**Fig. 2:**
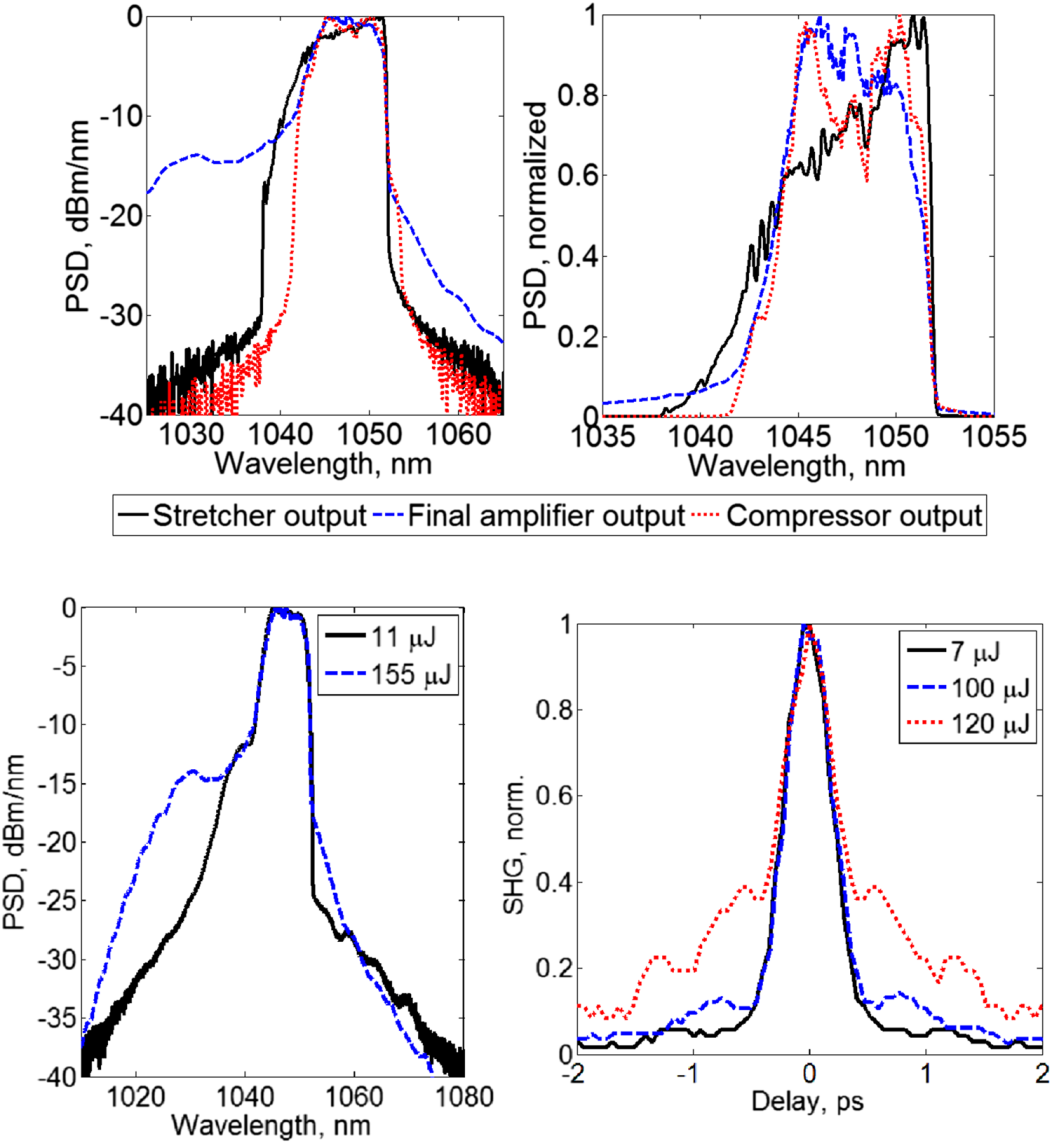
*Top left* Spectral evolution measured at three different points in the fiber CPA system: output of stretcher, output of final fiber amplifier, and output of compressor. (See legend below graphs.) *Top right* Spectrum on a linear scale. *Bottom left* Spectra measured at the output of the final amplifier for low and high amplifier output pulse energies. *Bottom right* Compressed pulse autocorrelation traces for increasing pulse energies

The top left and right plots of Fig. 2 show the spectral evolution throughout the system on logarithmic and linear scales. The data show the spectra (Yokogawa AQ6370C optical spectrum analyzer, resolution 0.05 nm) at the output of the stretcher, the output of the final amplifier and the output of the compressor. The 3-dB spectral widths were measured as 9 nm after the stretcher and then 8 nm after both the final amplifier and the compressor. The final edge-to-edge filter width was 12 nm. The hard edges on both the long- and short-wavelength sides of the spectrum are imposed by the spectral transmission window of the grating-based stretcher and compressor, which was approximately 14 nm. Gain narrowing was just 1 nm (spectral FWHM) for the entire amplifier chain, and the compressor output spectrum was reasonably flat considering the 3 dB width. This was achieved by tuning the gain of the amplifiers by appropriately choosing the Yb-fiber lengths and pump powers. The spectra at the output of the final amplifier for low and high pulse energy operation are shown in the bottom left plot of Fig. 2, highlighting the lack of fine-scale spectral modulation in particular as well as the low level of ASE from the final amplifier. The minimization of modulation was likely to be a result of using polarization maintaining (PM) fibers and polarizing isolators in the amplification chain because as well as ensuring thermal stability for the output polarization, this architecture avoids the formation of satellite pulses on the orthogonal polarization axis that can be amplified by nonlinear gain in the final fiber.

While optimizing the system, it was found that seeding the final amplifier with high input pulse energies from the first power amplifier led to increased self-phase modulation, which caused the pedestal on the autocorrelation traces to become significant at rather low output pulse energies. Therefore, the input to the final amplifier was reduced to the minimum possible commensurate with the ~14 dB output OSNR shown in Fig. 2. The bottom right plot shows the background-free SHG autocorrelation traces (APE PulseCheck 15) for compressed pulse energies of 7, 100, and 120 μJ (corresponding pulse energies at the final amplifier output: 11, 155, and 184 μJ), illustrating how nonlinear distortion limits the scope for further pulse energy increases. The pulse duration was 340 fs, assuming a Gaussian pulse shape. The transform-limited duration was 224 fs as calculated from the compressed pulse spectrum (Fig. 2 top row, red dotted line). The 100 μJ pulses were therefore 1.51 times transform limited. Third-order dispersion was optimized to partly compensate for the effects of self-phase modulation (SPM) by making small adjustments to the compressor grating input angle to maximize the autocorrelation peak and minimize the pedestal for the 100 μJ output pulses.

It has been known for a long time that once the B-integral in the amplifier of a CPA system reaches approximately 3 radians that the compressed pulse quality starts to degrade significantly as evidenced by a pedestal on the autocorrelation [22]. The figure therefore shows that nonlinearity in the final amplifier limited pulse energy scaling for this system, not energy storage in the fiber. The compressible pulse energy in fiber CPA is generally limited by the stretching ratio, which is set by the compressor grating width, or the design parameters of the amplifier fibers, e.g., the mode field diameter. The vast majority of the accumulated nonlinear phase occurs in the final amplifier. The calculated B-integral for the optical path to the launch into the final amplifier was 0.18π radians. For the final amplifier, the corresponding estimated B-integrals were 0.48π, 3.68π, and 4.26π radians for 7, 100, and 120 μJ compressed pulse energies, respectively. The peak power of the 1 ns pulses at the output of the final amplifier is 155 kW, which is well below the estimated Raman threshold power of 275 kW [23].

**Fig. 3:**
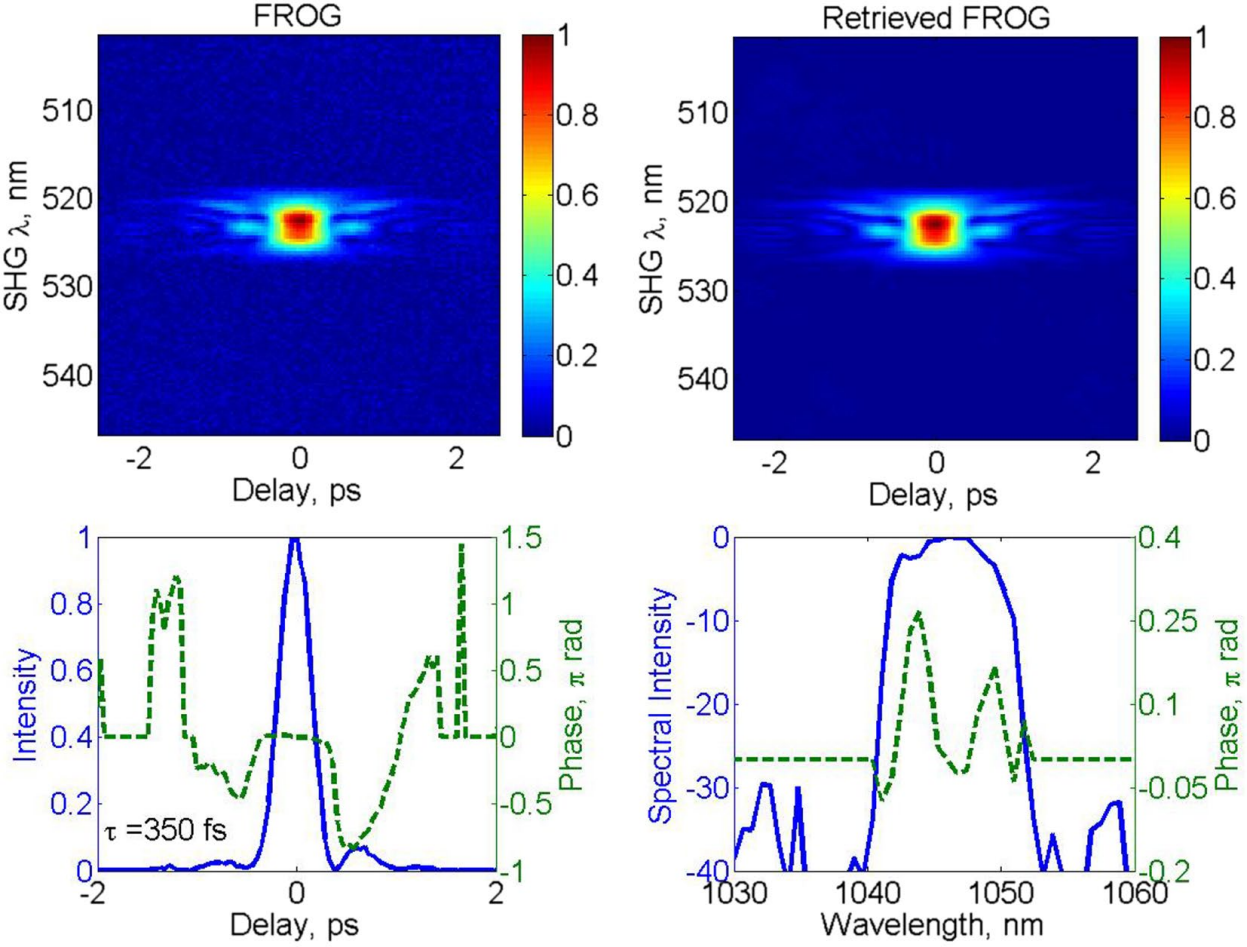
*Top row* Measured (*left*) and retrieved (*right*) SHG FROG traces. *Bottom row* Reconstructed pulse intensity and phase (*solid blue* and *dashed green lines*, respectively) in the temporal (*left*) and spectral (*right*) domains

The pulse duration of 340 fs estimated from the autocorrelation data was confirmed by direct measurement using SHG FROG (MesaPhotonics FROG Scan). Figure 3 (top left) shows the measured FROG traces for the 100 μJ output pulses. The raw data were taken with a temporal resolution of 20 fs and a spectral resolution of 0.08 nm. The FROG error measured 0.0049, and there was good agreement between the measured and retrieved FROG trace (top right). The bottom row of Fig. 3 shows the reconstructed intensity and temporal phase (left), and the reconstructed spectrum and spectral phase (right). The traces show a pulse duration of 350 fs at the intensity FWHM. The sign of the delay axis for this pulse retrieval is ambiguous due to the nature of SHG FROG. The phase across the central pulse peak is approximately flat, but the spectrograms show evidence of satellite pulses outside of the main pulse, which could be due to the nonlinear phase distortions as the autocorrelation pedestal is clearly reduced at lower pulse energies. The peak power of the pulses was calculated to be 240 MW from the FROG-reconstructed intensity profile. Active phase optimization as used in Ref. [19] is one option for further increasing the peak power by reducing the energy in the pulse pedestal by compensating for nonlinear distortion in the amplifier chain.

**Fig. 4:**
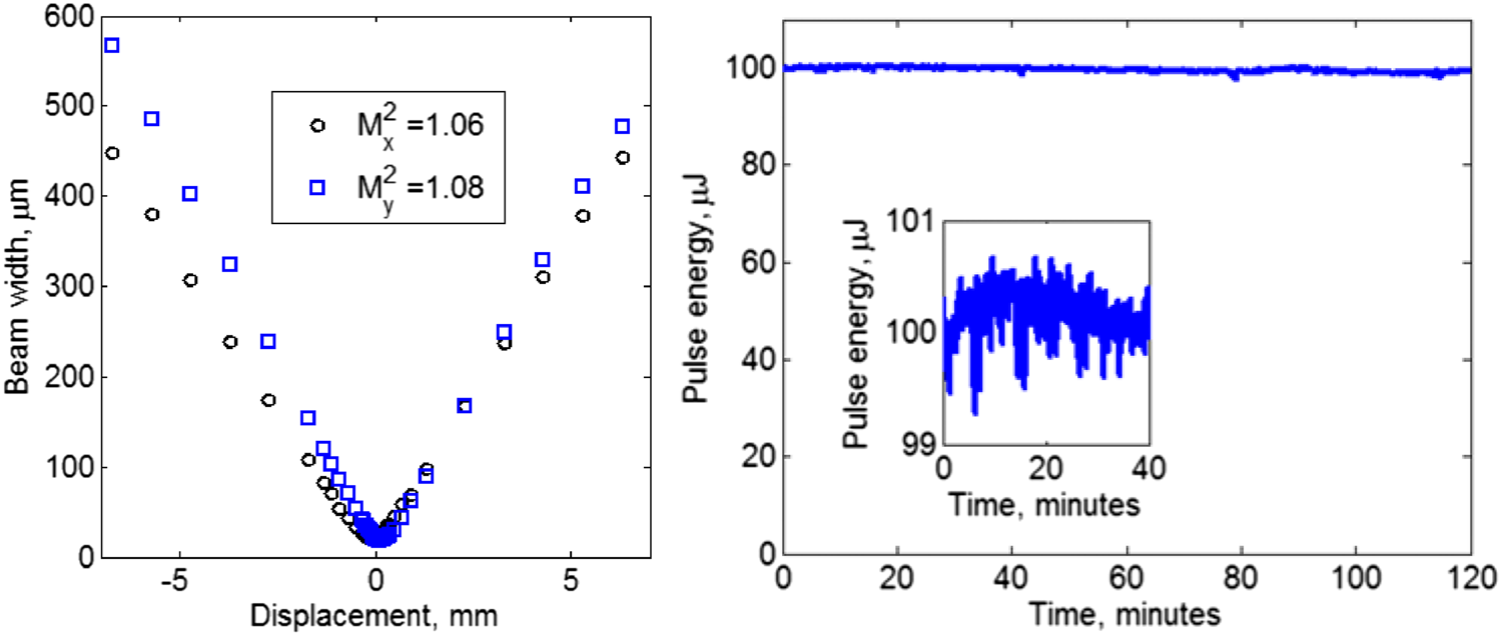
*Left* Beam caustics for the fiber CPA system, measured after the compressor. The average *M* ^2^ was 1.07. *Right* Long-term pulse energy stability measured after the compressor. The data have a standard deviation of 0.45 μJ about a mean pulse energy of 99.8 μJ. Inset: Power stability data plotted with just ±1% scale on the y-axis for the first 40 min after switching the final amplifier on

The beam caustics used for an *M* ^2^ calculation are shown to the left of Fig. 4 and were measured after the compressor. The spatial beam profile was almost diffraction limited, with an average *M* ^2^ value of 1.07 (1.06 and 1.08 in the *x*- and *y*-directions, respectively). The difference in the rate of beam divergence between the *x*- and *y*-data is the result of a slight difference in beam waist size (18.7 µm in *y*, 21.3 µm in *x*) as the *M* ^2^ value is the same for both. This ellipticity may be caused by the PCF used for the final amplifier, which is a polarizing design and hence may have an elliptical mode profile due to the stress-rods near the core. The cause of the slight asymmetry of the beam caustic around the focal position for the *y*-data was not identified, but may be the result of an imperfect lens alignment at the output of the final amplifier fiber, for example. However, the asymmetry is unlikely to result in any significant error in the measured *M*_*y*_^2^ value.

The right-hand plot of Fig. 4 shows the long-term stability of the system, measured over a 2 h period using a detector (ThorLabs S145C integrating sphere with InGaAs power sensor) with bandwidth set to 15 Hz so that there was averaging across approximately 100 pulses. The mean pulse energy was 99.8 μJ and a standard deviation of 0.45 μJ. The absolute minimum and maximum were 97.8 and 100.7 μJ, respectively.

Shot-to-shot pulse energy variation was measured at the output of the compressor using a 2 GHz detector (EOT ET3000 InGaAs) and comparing the peak of the diode output pulses on an oscilloscope. Over 30,000 samples it was found that the pulse energy had a standard deviation of <1%, with an absolute minimum and maximum pulse energy of 95 and 103 μJ.

## High-harmonic EUV generation

### System

**Fig. 5:**
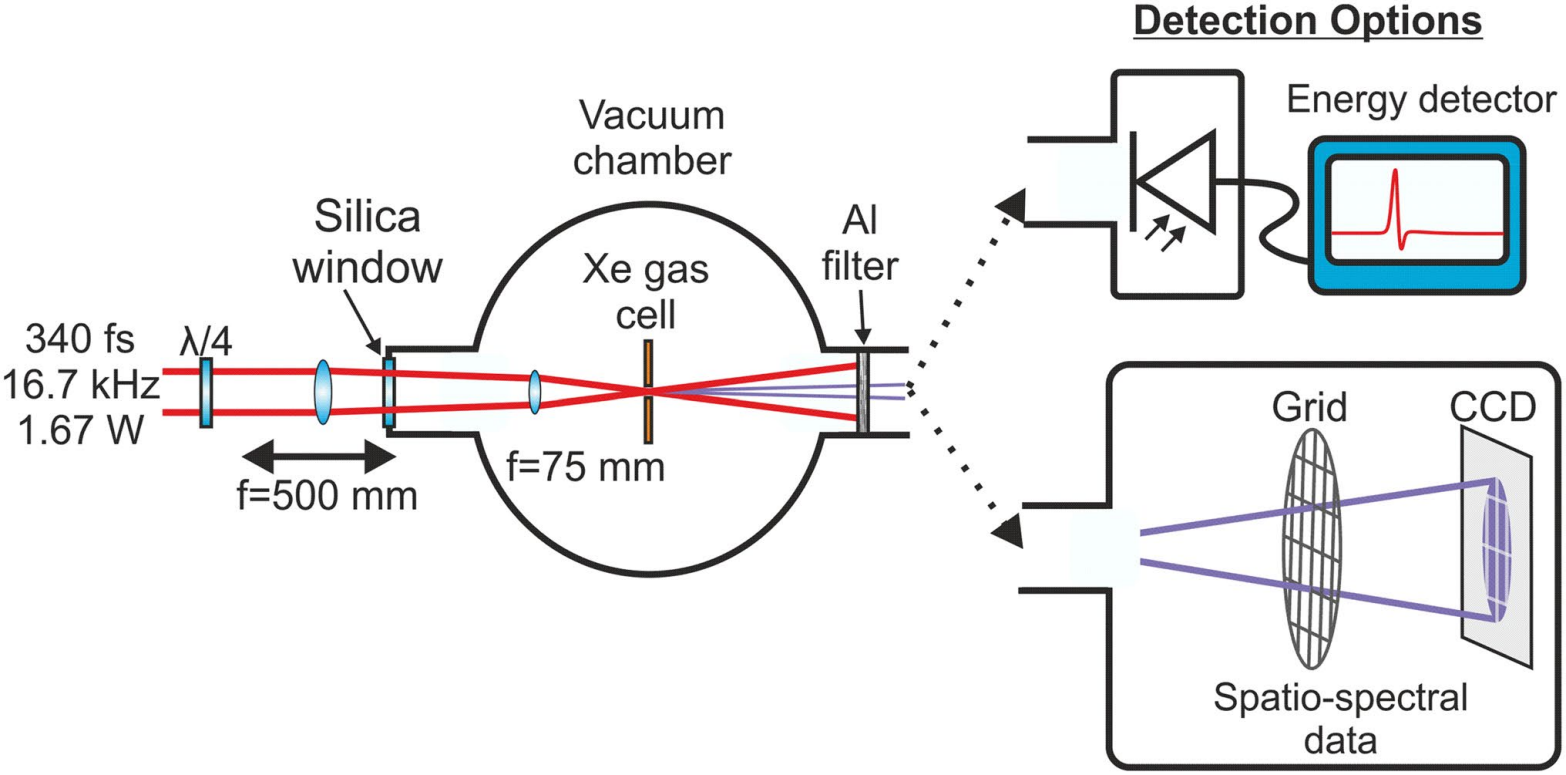
Schematic of the EUV generation and detection system used with the fiber CPA system. The EUV diagnostics were housed in separate vacuum chambers that could be attached to the main EUV generation chamber. The energy detector was an Al-coated EUV photodiode with a low noise amplifier. The spectrum was reconstructed from the diffraction pattern from a grid recorded on a CCD camera

To demonstrate the suitability of the system for EUV generation, a vacuum chamber and diagnostics were set up with a low pressure xenon gas cell positioned at the focus as shown schematically in Fig. 5 [24]. This test-bed setup was used to measure conversion efficiency which in our case was recorded using an EUV optimized photodiode. The spectrum was measured using the method outlined in Refs. [25, 26].

When using a gas cell and a small focal spot, phase matching becomes crucial because the long absorption length of the cell can overcome the benefits of an extended interaction region. To overcome this, the coherence length must be increased. This can be done in the tight-focusing regime by balancing the Guoy phase shift with the phase matching contribution from the atomic phase, gas pressure, and ionization fraction. In practice, this is achieved by longitudinally translating the focus through the gas cell to optimize the XUV flux.

The focus of the IR beam was designed to maximize the volume over which tunnel ionization dominated. It was found that a 70-mm focal length was appropriate which, if a single lens were used, would require the lens to be mounted inside the chamber, making axial adjustments difficult. Therefore, in order to scan the focus axially through the gas cell to achieve the required balance between the atomic and Gouy phase for optimum EUV generation a telescope was constructed from a 500-mm plano-convex lens positioned outside of the chamber and separated from a 75-mm best-form lens inside the chamber. The external 500-mm lens was translated longitudinally to shift the focus through the gas cell. The effective focal length of this lens setup was 6.78 cm. Experimentally, we swept the focal spot axially through the gas cell while monitoring the XUV diode output to determine the position producing the maximum measurable flux. The physics is complicated because this position trades off generation efficiency (optimum in denser regions of the gas) against subsequent reabsorption as the XUV transits through the background levels of Xe to the detector. Using ABCD matrix calculations, we estimated the average focal spot size over x and y was approximately 5.1 μm at the intensity FWHM suggesting a Rayleigh length of 78 μm. The generation region was clearly visible from blue emission from the Xe plasma recombination that occurs between the driving laser pulses, and the optimum signal was measured when the generation region was approximately 0.5 mm after the gas cell.

The standard technique of using the strong differential between the EUV and visible/IR transmission characteristics of an aluminum filter (200 nm thick) was employed to separate the residual laser power from the EUV at the output. The EUV Al transmission band is from ~17 to 80 nm [27]. An Al-coated EUV photodiode was then used to detect the integrated energy in the EUV pulses. A low noise amplifier was included to boost the diode output signal and averaging over 256 samples on an oscilloscope produced a noise floor of −1 mV. This enabled straightforward and fast optimization of the Xe pressure and the axial position of the focus relative to the Xe gas cell.

### EUV generation results

**Fig. 6:**
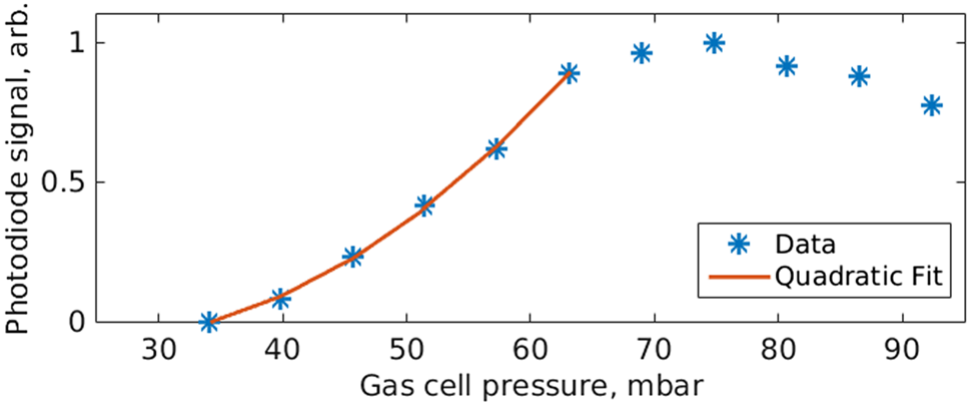
Pressure dependence of the XUV photodiode signal (normalized)

**Fig. 7:**
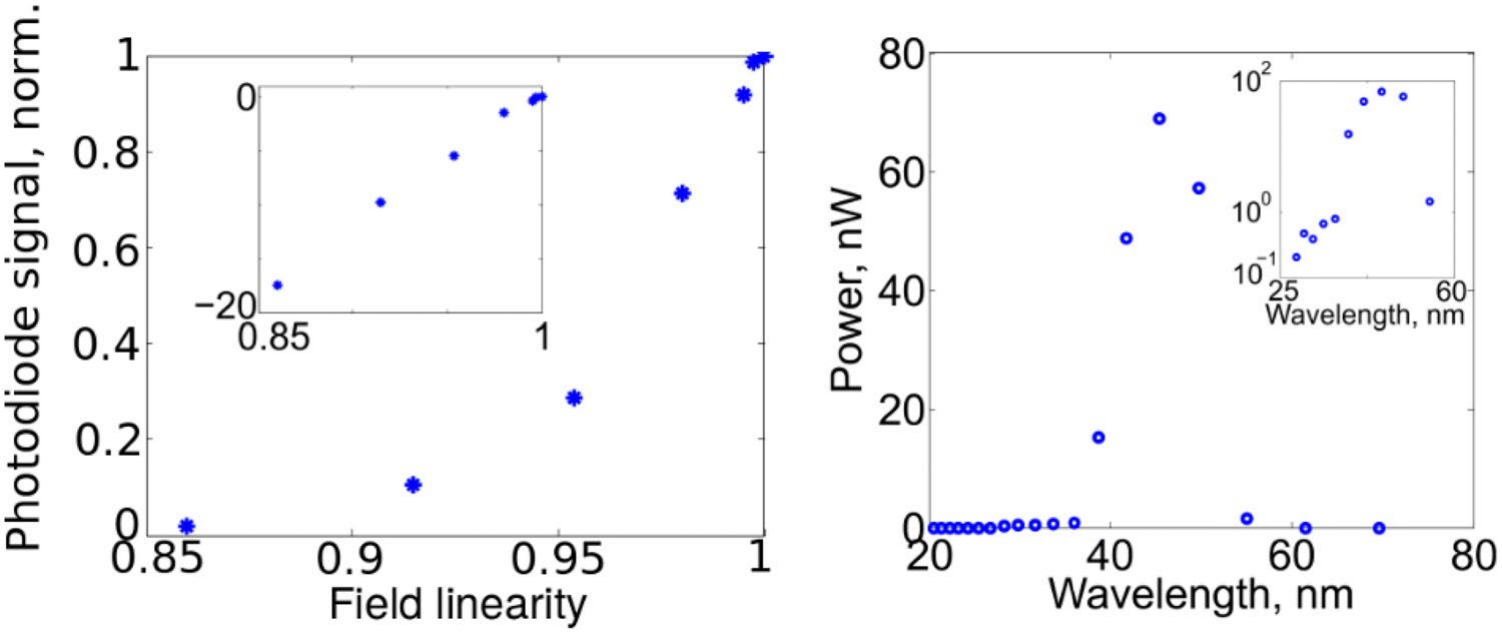
*Left* EUV photodiode signal as the driving laser field polarization is changed from linear (linearity = 1) to elliptical (linearity <1). Inset: Normalized photodiode signal in dB. *Right* High-harmonic spectrum generated by the fiber CPA system. The powers have been scaled to their lowest estimated values. The inset shows the power spectrum of the harmonics on a logarithmic scale

The EUV flux was maximized when the focus was positioned ~0.5 mm behind the gas cell, which was filled with 75 mbar of Xe. Figure 6 shows how the XUV photodiode signal varied with increasing gas pressure. The focal position was optimized for highest flux for each pressure used. As expected, the flux increases quadratically as the Xe cell pressure is increased. However, above 75 mbar, reabsorption dominates and reduces the detectable XUV signal. The gas cell had an interior depth of approximately 0.5 mm. It was confirmed that the measurements were recording EUV flux rather than residual driving laser power by observing the dependence of EUV flux on the polarization state of the driving laser field. The results are shown in the left plot in Fig. 7, in which the linearity is defined on the interval [0;1] for completely circular and completely linear polarization, respectively. A maximum extinction of 18 dB was measured before the signal dropped below the noise floor for a reduction in linearity of just 0.14, thus confirming that the observed signal was indeed produced by HHG.

The EUV flux was calculated from the photodiode signal using Eq. (1), below:

1 $$n_{\text{ph/s}} = \frac{VC}{{9200q_{e} \delta }}f_{\text{rep}}$$

where, *n*_ph/s_ is the number of photons generated per second, *V* is the voltage read from the oscilloscope, *C* = 2 nF is the capacitance of the photodiode, *q*_*e*_ is the electron charge, and *f*_rep_ = 16.7 kHz is the repetition rate. Equation 1 uses the average quantum yield of the photodiode, specified by the manufacturer as two electrons per photon, and the manufacturer’s specification of 4600 gain for the preamplifier. The parameter *δ* is the overall transmission of the residual Xe gas between the gas cell and the detector, the aluminum filter, aluminum photodiode coating and the resulting three aluminum oxide layers that form on each coating surface with total thickness of ~15 nm (assuming 5 nm for each exposed surface of aluminum—the typical self-terminating oxide thickness on exposed surfaces [28]). The two Al filters used to separate the XUV from the residual infrared had a total thickness of 350 nm and the approximate total thickness of the self-terminating Al_2_O_3_ layers on the exposed faces of the filters was 15 nm. Hence, using materials data for the 40-nm wavelength region from the Center for X-Ray Optics Database [29], we estimated that the filter transmission was 4%. The Xe transmission was similarly calculated to be 56% through a 13-cm path length at a background pressure of 0.08 mbar. The estimated thickness of the Al_2_O_3_ layers is the dominant uncertainty in the attenuation and hence in our estimated XUV flux, and we allow for a factor of two in the attenuation coefficient, giving an approximate XUV transmission of 2.5–5% with central value of 4%.

We found that optimizing the XUV flux with an iris introduced additional loss which reduced the XUV flux monotonically as the iris was closed. Therefore, the FWHM beam diameter at the compressor output was iteratively adjusted using the telescope at the output of the final amplifier to optimize the phase matching and hence XUV flux. The XUV photodiode voltage was used to provide real-time feedback. The axial focal position and gas cell backing pressure were also scanned for each iteration to find a globally optimal set of XUV generation parameters. A FWHM beam diameter at the CPA system output of 3.3 mm was found to give the best generation efficiency and the pressure scan data recorded with this beam diameter are shown in the figure above.

Using the parameters in Eq. (1), the maximum flux was calculated to be 0.5–1 × 10^11^ photons/s, which corresponds to 200–400 nW of EUV power within the Al transmission band. The right-hand plot of Fig. 7 shows the EUV spectrum on linear and log scales measured using the method outlined in [25, 26]. The peak wavelength is 45 nm, and the shortest wavelength is approximately 27 nm. This spectrum was also used when calculating the EUV power by taking the relative harmonic amplitudes and photon energies into account.

## Discussion

To establish a standard measure for comparison between the EUV generation efficiency of the fiber CPA system presented here and others in the literature a metric was defined using the ratio of EUV output power to input IR power (*η* = P_EUV_/P_IR_). This metric, *η*, was chosen as it presents an intuitive measure of conversion efficiency from the perspective of an end user of these systems. Table 1 provides details of various fiber CPA systems reported in the literature and their corresponding EUV generation efficiencies, chosen due to the similarities between the reported systems and that presented here. All EUV powers were estimated from the data given in each reference where the value was not stated directly. Where nonlinear pulse compression was used in a separate stage after the fiber CPA the efficiency was calculated using the IR power before the nonlinear compression stage to give the efficiency of energy transfer from the laser to the EUV.

**Table 1:** Review of the performance recently reported for fiber CPA systems in HHG experiments. The results for the fiber CPA system reported here are summarized in the final row. EUV powers and photon fluxes have been estimated from the data where they are not given

Reference	IR power (W)	Pulse duration (fs)	EUV power (nW)	*η* = P_EUV_/P_IR_ (nW/W)
[14]	10	800	1–100	1–10
[15]	80	500–45	9200	115
[30]	50	500	4000	80
[31]	0.7	7	11	57
[34]	~165	29	600,000	~3600
This work	1.67	350	300 ± 100	180 ± 60

Cabasse et al. [30] detect 10^12^ photons/s with a driving laser power of 50 W. This measurement is made over harmonics 15, 17, 19, 21 of a 1030-nm driving laser, corresponding to the following photon energies in each harmonic: 2.89 × 10^−18^ J (15th), 3.28 × 10^−18^ J (17th), 3.67 × 10^−18^ J (19th), and 4.05 × 10^−18^ J (21st). At 10^12^ ph/s, we estimate their average XUV power to be approximately 3500–4000 nW (an average of 1 µW per harmonic, which agrees with their abstract). Using our metric for conversion efficiency with their stated average input laser power, this corresponds to a maximum of 4000 nW of XUV power for 50-W input power, or 80 nW/W, which is lower than achieved here. This is expected because the authors report the same pulse energy as here but a longer (>500 fs) pulse duration [30].

In the tight-focusing regime, a low pulse energy, 100 kHz, Ti:Sapphire system producing 45 fs, 7 uJ pulses produced 3 × 10^9^ photons/s in the 15th harmonic in Xe, corresponding to approximately 11 nW in this harmonic [31]. Assuming an equal power 13th harmonic (not shown in the data) and including the weaker 17th and 19th harmonics shown, we estimate an integrated XUV power of 40 nW which, for 0.7 W of input power, gives 57 nW/W conversion, a value significantly below that achieved here.

The calculated ionization fraction for a 100 µJ, 340 fs pulse reaches 100% before the pulse peak. The resulting phase matching contribution from the generated plasma cannot be overcome, and as such HHG is restricted to the leading edge of the pulse for the system presented here. This effect is greatly reduced when shorter pulse durations are used as there are fewer optical cycles in the pulse where ionization can take place, leading to a low ionization fraction and improved phase matching over the entire pulse duration. Shorter pulse durations could be achieved with our system by using nonlinear compression alongside further energy scaling to overcome the additional compressor loss while still giving sufficient output intensity for driving HHG. This represents an attractive route for further improving the generation efficiency of the system. This has been achieved in other systems using SPM in a gas-filled capillary [32] or in a Kagome fiber [33] followed by chirped mirrors for dispersion compensation. This method has been used in conjunction with coherently combined fiber amplifiers to create significantly higher pulse energies, and it has been shown that it is possible to increase the generation efficiency by not only reducing the ionization fraction but also by enabling a much larger spot size to be used. This permits a larger phase matching interaction volume, thus resulting in an increase in the number of atoms involved in the HHG process for a given pressure as shown by the efficiency metric for the system in Ref. [34]. This could also be a way of obtaining XUV down to the absorption limit of ~21 nm (set by the Al filters and background Xe), whereas the high ionization in our system limits the minimum wavelength to 27 nm. However, both the nonlinear compressor and the use of parallel and coherently combined final amplifiers in the case of Ref. [34] result in additional complexity in comparison with single-fiber systems. A careful trade-off between performance and practicality or cost must therefore be considered for each specific application.

For the other systems that use a single-fiber amplifier channel and considering both linearly and nonlinearly compressed results, it is seen that *η* = 1–115 nW/W in the literature [14, 15]. For the system presented here, *η* = 180 ± 60 nW/W. We show that this coversion efficiency is approximately 1.17 times greater than comparable systems using a single rod-type fiber, with an upper limit on this estimate of 1.56 times and, therefore, indicates that this system demonstrates state-of-the-art conversion efficiency for single-fiber CPA architectures including both linear and nonlinear compression. This is attributed to the excellent driving laser stability, diffraction-limited beam quality, and maximized tunnel ionization volume.

A simpler route to improving the EUV generation efficiency beyond our 180 nW/W without involving the use of coherently combined parallel amplifier channels would be to follow the example outlined in Ref. [35], where gas pressures exceeding 8 bar were used to better overcome the effect of the Gouy shift on the phase matching when using a tight focus. This method was not used here due to the limited pumping speed of the vacuum equipment in our laboratory, but its application simply requires investment in higher capacity vacuum pumps.

For the system presented here, additional technical benefits to system stability could be achieved by fully fiberizing the CPA system to eliminate potential alignment drifts associated with the remaining free-space optics in the amplifier chain. For example, using a chirped-fiber Bragg grating (CFBG) [12] and fiberized circulator for the stretcher (or a specially designed combination of stretcher fibers with dispersion matched to the compressor as done in Ref. [36]) would enable complete integration at the system front end. A fiberized final amplifier has become possible following the launch of a commercialized pump combiner spliced to the NKT DC-200/40-PZ-Yb-fiber in the final amplifier and employing that in our system could improve the stability of the pump and signal coupling. In addition, using higher power pump diodes to scale repetition rate would directly increase the EUV average power, making better use of the high generation efficiency. Newer, more efficient, grating designs [37] allow for higher compressor throughput and thus would reduce the pulse energy required from the final amplifier for a fixed compressor output. The associated reduction in the B-integral would lead to cleaner compressed 100 μJ pulses and the improved concentration of energy in the pulse peak would increase the peak power, thus enabling looser focussing and improved HHG flux.

## Conclusion

We have reported a detailed account of the development of a stable, compact, and power scalable polarization maintaining Yb-fiber CPA system based on bendable fiber technology, giving 350-fs diffraction-limited pulses with peak powers of 240 MW. The system was used for high-harmonic generation, and the resulting EUV flux was optimized by basing the focal geometry on calculations of the focal volume over which tunnel ionization dominates. In comparison with previously published reports of similar systems with single-fiber final amplifier channels the conversion efficiency from IR to EUV wavelength ranges was better than the current state of the art. Future applications could include coherent diffractive imaging in the extreme ultraviolet, and high-harmonic spectroscopy. The CPA system reported here therefore provides a stable, compact polarization maintaining fiber platform for frequency conversion to the EUV via high-harmonic generation with high EUV conversion efficiency.
